# Recent Advancements in the Synthesis of Covalent Triazine Frameworks for Energy and Environmental Applications

**DOI:** 10.3390/polym11010031

**Published:** 2018-12-26

**Authors:** Ying Zhang, Shangbin Jin

**Affiliations:** School of Chemistry and Chemical Engineering, Huazhong University of Science and Technology, Wuhan 430074, China; yingzhang0427@163.com

**Keywords:** covalent triazine frameworks, synthesis, application, energy, environment

## Abstract

Covalent triazine frameworks (CTFs) are a unique type of porous materials, comprised of triazine units. Owing to the strong linkage of triazine, the most important advantage of CTFs lies in their high chemical and thermal stabilities and high nitrogen content as compared to other porous organic polymers (POPs). Therefore, CTFs are one of the most promising materials for practical applications. Much research has been devoted to developing new methods to synthesize CTFs and explore their potential applications. Nowadays, energy and environmental issues have attracted enormous attention. CTFs are particular promising for energy- and environment-related applications, due to their nitrogen-rich scaffold and robust structure. Here, we selected some typical examples and reviewed recent advancements in the synthesis of CTFs and their applications in gas adsorption, separation, and catalysis in relation to environment and energy issues.

## 1. Introduction

Porous organic polymers (POPs) are a new class of nanoporous materials which were discovered in recent decades. Their highly porous nature and covalent bonding structures endow them with large surface areas and high stability. These characteristics make them useful in a number of advanced functional applications, reason for which they are receiving large research attention nowadays in materials science [1,2]. POPs can be divided into several representative subtypes, namely, conjugated microporous polymers (CMPs) [3], covalent organic frameworks (COFs) [4,5], covalent triazine frameworks (CTFs) [6], porous aromatic frameworks (PAFs) [7], hypercrosslinked polymers (HCPs) [8], and polymer of intri nsic microporosities (PIMs) [9]. Among them, CTFs, structurally comprised of triazine units, are a unique type of porous materials. The aromatic C=N bond in triazine units endows CTFs with high stability and allows their applications in harsh conditions. Structurally, CTFs present as either crystalline or amorphous materials. Currently, most CTFs are amorphous, because of the high stability of the triazine linkage and low reversibility of the polymerization procedure. Several methods have been developed for the synthesis of CTFs, which are ionothermal synthesis [6], superacid catalysis [10], transition metal coupling reactions [11], and polycondensation method [12]. CTFs are reported to be practicable in a number of functional applications, such as gas adsorption and storage [13,14,15,16,17,18,19,20,21,22,23,24,25,26,27,28,29], heterogeneous catalysis [14,30,31,32,33,34,35,36,37,38,39,40,41,42,43], energy storage and conversion [44,45,46,47], photocatalysis [12,48,49,50,51,52]. In this review, we selected typical examples of CTFs reported in the literature and reviewed the recent development in their synthesis and application in gas adsorption and catalysis in relation to energy and environment issues.

## 2. Structural Characteristic and Synthesis Routes

**Figure 1:**
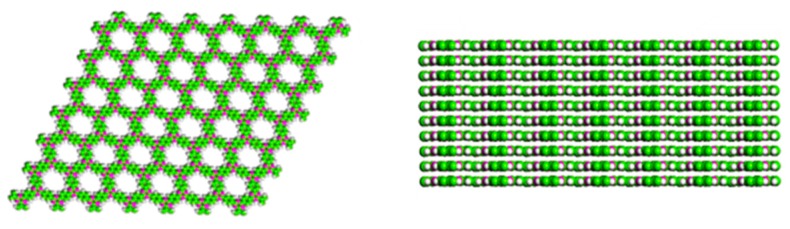
Top-down and side view of covalent triazine frameworks (CTF)-1, with a crystalline ordered structure [6].

CTFs are comprised of triazine units; therefore, they are structurally nitrogen-abundant materials. Such kind of structure endows them with properties useful in many promising applications (Figure 1). The triazine units are very stable, and therefore the materials can endure harsh conditions such as acid or basic conditions, which cannot be tolerated by other porous organic polymers. CTFs bear very highly conjugated structures, which confer semiconductive properties. The structure of CTFs can be either crystalline or amorphous. It is desirable to achieve high crystalline CTFs, however, most of the CTFs currently available are amorphous. The difficulty in obtaining crystallinities lies in the low reversibility of the linkage. 

**Figure 2:**
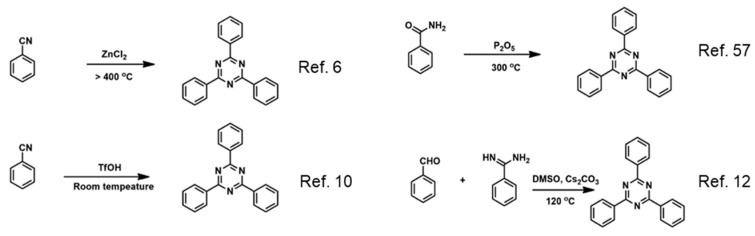
Typical reactions for the synthesis of CTFs.

Limited methods have been developed for CTFs synthesis (Figure 2). The first method was reported by Thomas et al., who used ionothermal conditions to synthesize CTFs from nitrile monomers [6]. This method was conducted at high temperature, usually over 400 °C, and needed to be carried out in a sealed ampoule tube. These very harsh conditions do not allow large-scale synthesis. However, CTFs synthesized by ionothermal condition can achieve a very high surface area, which can be attributed to the template effect of the Lewis catalyst and partial carbonization of the materials. The nitrogen-rich frameworks can also be achieved through the introduction of nitrogen-rich building blocks (i.e., bipyridine [53], porphyin [54], phthalazinone [28], acetylacetone [55], etc.), which are good supports for various metal nanoparticles and metal complexes. This is very important for the applications of CTFs in catalysis and adsorption. Later, Cooper et al. developed a method for CTF synthesis requiring mild conditions, in which trifluorosulfonic acid was employed as a catalyst to polymerize nitrile monomers, allowing CTF synthesis at room temperature or in microwave conditions [10]. Along this superacid method, recently, Xu et al. reported the use of interfacial conditions by which CTFs were synthesized with layered structures and crystallinity [56]. The above methods are all based on the trimerization of nitrile monomers. CTFs from other monomers are less developed. Recently, we reported a polycondensation reaction from aldehyde and amidine, which is a useful method to synthesize CTFs at relatively low temperature [12]. The CTFs prepared by this polycondenstaion method avoided partial carbonization and exhibited semiconductive structures and high surface areas. This method is also expected to lead to CTFs with more structural diversity, since the polycondensation involves two types of monomers, broadening the possibilities of structural design. Another method based on amide monomers was also reported, where P_2_O_5_ was used as a catalyst and reaction medium. However, the polymerization was performed at a rather high temperature [57]. Microwave has also been used to synthesize CTFs. Wang et al. reported a microwave-enhanced ionothermal synthesis method to prepare CTFs with surface area up to 2390 m ^2^ g^−1^ [58]. Recently, Tang et al. also reported that a high crystalline CTF-1 was prepared by the microwave method in a very short term, just tens of minutes [59]. Therefore, more and more methods are emerging for CTF synthesis.

## 3. Energy- and Environment-Related Applications

The large surface area and semiconductive structures provide CTFs with an enormous potential for applications in many areas, such as gas adsorption and storage, heterogenerous catalysis, energy storage and conversion, and photocatalysis. Combining these properties with a high stability, CTFs appear as a potential material for practical applications in the field of energy and environment. Here, we focus on the recent advances in the application of CTFs in areas related to energy and environment. 

### 3.1. Hydrogen Storage and CO_2_ Capture

Hydrogen is the most promising energy source to achieve a sustainable society. However, hydrogen storage is the critical step for the practical applications of hydrogen energy. Therefore, the development of materials for hydrogen storage is the most important task in the field. On the other hand, CO_2_ is the most typical greenhouse gas, which is due to the usage of the fossil fuels. Thus, it is also urgent to decrease CO_2_ levels in the atmosphere. One solution to alleviate these problems is the employment of porous organic polymers to capture and storage these gases. 

#### 3.1.1. Hydrogen Storage

CTFs are particularly promising for the adsorption and storage of hydrogen (Table 1). Thomas et al. originally presented the synthesis of CTFs and investigated the applications of CTFs in hydrogen storage [6]. They showed that the CTF synthesized from 4,4-dicycnobiphenyl (DCBP) monomers has a high surface area of 2475 m^2^ g^−1^, which can storage 1.55 wt % hydrogen in the condition of 1 bar and 77 K [6]. The performance is comparable to that of many other porous materials. Han et al. reported that hierarchical traizine-based porous carbon is a promising material for hydrogen storage. A surface aera of 1200 m^2^ g^−1^ can be obtained, and the hydrogen uptake can reach 2.34 wt % at 77 K and 1 bar. They concluded that hierarchical porous structures can be good for small-molecule diffusion and thus benefit gas uptake [60]. Janiak et al. synthesized porous covalent triazine-based organic frameworks (PCTFs) from a supramolecular tetranitrile building block, which resulted in a high surface area of 2235 m^2^ g^−1^ and, more importantly, to hydrogen storage reaching a high capacitance of 1.86 wt % at 77 K and 1 bar [61]. Lotsch et al. reported a fluorine-based CTF (fl-CTF-400) which could uptake 1.95 wt %, which is higher than the uptake of CTF-1 (1.55 wt %), PCTF (1.86 wt %), and PCTF-2 (0.9 wt %) [62]. They further increased the pressure up to 20 bar, achieving an adsorption of 4.36 wt % [62]. In 2017, Coskun et al. developed a chemical activation approach to enhance the textural property of CTF. They found that the activated CTF-1 at 700 °C could result in a significantly enhanced surface area up to 2367 m^2^g^−1^, with a hydrogen uptake ability up to 2.46 wt % at 77 K and 1 bar [26]. These reports showed that it is important to achieve high surface area and suitable pore size of CTFs for hydrogen storage. However, most of the reported CTFs are focused on hydrogen adsorption, while the hydrogen desorption process is also important but is less studied.

Theoretical calculation is also a powerful tool to investigate the application of CTFs for hydrogen storage. In 2013, Yu et al. showed that CTF-1 can storage hydrogen by introducing various metal, i.e., Li, Na, K, Mg, Ca, through ab initio density functional calculation [63]. The theoretical calculation demonstrated that Li, Na, K, and Ca can be introduced into CTF-1 as a result of charge transfer interaction between the introduced atoms and the supported framework. It was shown that a highest uptake of 12.3 wt % can be achieved in a CTF–Li complex, theoretically. He et al. further showed that transition metals, such as Sc, Ti, V, Cr, Mn, can be supported in CTFs by using ab initio density functional theory (DFT) calculation [64]. Their calculation results showed that the absorbed metals can exist without forming clusters. Interestingly, they found that V, Cr, and Mn are not proper metals because of the large binding energy for hydrogen, but Sc and Ti are promising metals for hydrogen uptake at ambient conditions. These works showed that CTFs are potential materials for hydrogen storage by incorporation of metals in the frameworks.

#### 3.1.2. Carbon Dioxide Capture

Cooper et al. investigated the carbon dioxide adsorption of CTFs prepared by the catalysis of superacids. They revealed an exceptional adsorption capacity up to 4.17 mmol g^−1^ for CTFs [10]. In their work, P6M had the highest adsorption capacity for carbon dioxide even though it was not the polymer with the highest Brunauer-Emmett-Teller (BET) surface area. They also studied the adsorption selectivity over nitrogen, for which P1M had the highest CO_2_/N_2_ selectivity of 31.2, although it had the lowest surface area. Janiak et al. reported a new adamantine-based CTF in 2013, which was prepared in high yields using either ZnCl_2_ or CF_3_SO_3_H as catalysts. They optimized the synthetic conditions and Lewis acid catalysis conditions. Under the optimized conditions, the highest BET surface of 2235 m^2^ g^−1^ and an adsorption of 73 cm^3^ g^−1^ CO_2_ were obtained. They also found that the CTF has high gas adsorption selectivity corresponding to 41:1 for CO_2_/N_2_ and 7:1 for CO_2_/CH_4_ [65]. Han et al. designed perfluorinated CTFs (FCTF-1) and showed the introduction of fluorine has an obvious positive effect on CO_2_ adsorption and separation [66]. The FCTF-1 can uptake 1.76 mmol g^−1^ CO_2_ at low pressure (0.1 bar) and 273 K. They also found that FCTF-1 has a high selectivity of 77 for CO_2_/N_2_ separation, which is due to its ultramicropores. Interestingly, due to the hydrophobic nature of fluorine, the TCTF-1 is tolerant to water in gas separation. The good gas adsorption ability of CTFs is partially due to the high nitrogen content. Therefore, it is critical to introduce nitrogen in the framework of CTFs. Indeed, Lotsch et al. introduced a series of nitrogen atoms in the CTFs and achieved high CO_2_ adsorption and selectivity [67]. They showed that bipyridine–CTFs have the highest CO_2_ uptake up to 5.58 mmol g^−1^ at 273 K and have a high CO_2_/N_2_ selectivity up to 189. They revealed that microporosity was the main factor for high CO_2_ uptake, and the N content was the main contributor for high selectivity. Janiak et al. developed a new strategy to synthesize CTFs with two linkers [68], which showed better CO_2_ adsorption and separation performance than the individual linkers [69]. The higher CO_2_ adsorption of mixed-nitrile CTFs was attributed to the higher micropore content and microporous volume. Tuci et al. developed a new *N*-doped CTFs with microporosity (CTF-py) showing excellent CO_2_ adsorption ability at 0.1 bar, with 2.03 and 1.12 mmol g^−1^ at 273 K and 298 K, respectively, which is better than that of TCTF-1. They also showed high CO_2_ adsorption at 1 bar, with 5.97 and 4.22 mmol g^−1^ at 273 K and 298 K, respectively [70]. Voort et al. designed N-heteroaromatic structures as polymerization monomers for CTFs and synthesized CTFs by optimization of the catalyst and monomer ratios and polymerization temperatures. They found that CTF-20-400 had the highest CO_2_ uptake ability in the series, with a capacity of 3.48 mmol g^−1^ at 1 bar and 273 K [71]. Yu et al. also introduced N-heteroacyclic units (carbazole) for CTFs, which can capture and fix CO_2_. They synthesized CTF–CSUs (CSU: Central South University) using carbazole nitriles monomers using a bottom-up strategy, with a highest BET surface area of 982 m^2^ g^−1^ and a good CO_2_ uptake ability of 12.9 wt % at 273 K and 1 bar. They further showed that CO_2_ can be fixed into cyclic carbonate with high yields [72]. Voort et al. recent reported an acetylacetone CTF (acac-CTF), introducing dual N and O sites in the CTFs. The acac-CTF can achieve a BET surface area as high as 1626 m^2^g^−1^ and can exhibit a high CO_2_ upake of 3.3 mmol g^−1^ at 273 K and 1 bar. Good selectivity towards N_2_ was also shown, with a CO_2_/N_2_ up to 46 at 298 K [55]. On the other hand, porous carbon based on CTFs was also developed for CO_2_ adsorption. For example, Han et al. reported a series of triazine-based porous carbon materials (TPCs) which were constructed from fluorinated aromatic nitrile building blocks using the ionothermal synthesis method, resulting in triaizne-based porous carbon materials. The highest achievable BET surface area was 1940 m^2^g^−1^, and the best CO_2_ adsorption ability could be up to 4.9 mmol g^−1^ at 273 K, 1.0 bar [73].

Besides heteroatom introduction, adoption of charge units in the frameworks can also be a promising strategy to capture and fix CO_2_. To this end, Coskun et al. designed a dicationic viologen structure as a building block for CTFs and prepared CTFs at ionothermal conditions. The highest surface area reached was up to 1247 m^2^ g^−1^, and the CO_2_ uptake could reach 133 mmol g^−1^ at 1 bar and 273 K. More interestingly, CO_2_ can be fixed to cyclic carbonates [27]. There are also some other reports of triazine-based porous materials, such as triaizne-based covalent organic framework (COF) with BET surface area of 609 m^2^ g^−1^, which can uptake 57.07 wt % CO_2_ at 273 K and 5 bar [74].

These reports showed that CTFs can be well designed (Table 2). We can either introduce a series of heteroatoms or design the structures of the monomers to achieve higher uptake abilities. These traits endow CTFs with promising potentials in CO_2_ capture and storage.

### 3.2. Photocatalytic Water Splitting and Carbon Dioxide Reduction

#### 3.2.1. Photocatalytic Water Splitting

CTFs have highly conjugated structures, which can be tunable through structural design and synthesis. Their semiconductive structure and the energy levels can be finely tuned. In addition, they mostly have good absorption in the visible light. Therefore, CTFs are promising photocatalysts for various applications.

Zhao et al. firstly conducted the theoretical calculation of CTFs using the first-principle method. They foresaw that CTFs having layered structures are promising photocatalyts for water splitting [75]. As mentioned above, CTFs synthesized by the ionothermal method are generally not suitable for photocatalysis, while CTFs prepared by the superacid catalysis method can avoid carbonization and remain the inherent band gap of CTFs. Indeed, Wu et al. demonstrated that CTF-T1 prepared by trifluorosulfonic acid has good photocatalytic hydrogen evolution performance in the visible light; however, the photocatalytic hydrogen evolution is low and it is only comparable with that of bulk carbon nitride [48]. Heteroatom doping can also improve the photocatalytic performance. Su et al. reported that sulfur can be doped in CTF-1 and showed greatly enhanced hydrogen evolution rates up to 2000 μmol h^−1^ g^−1^ [76]. Wu et al. further showed that CTF can be hybridized with MoS_2_ and achieved an enhanced photocatalytic hydrogen evolution performance, which was explained by the enhanced interfacial charge transfer and separation [51]. Although the high-temperature ionothermal method may cause partial carbonization of CTF, Lotsch et al. showed that CTF oligomers prepared at 300 °C exhibited good photocatalytic hydrogen evolution [49]. Thomas et al. recently further showed that CTF prepared by the ionothermal method also present good photocatalytic performance for hydrogen evolution [77]. The key for the success lies in the control of the reaction time in the ionothermal stage, which could reduce the carbonization degree of the materials. 

Recently, Tang et al. reported a microwave-assisted synthetic method to prepare CTF-1, which showed high crystallinity [59]. The resulted materials were reported to exhibit good hydrogen evolutions as high as 5500 μmol h^−1^ g^−1^. They also reported high quantum yields, which could be up to 6% at 420 nm. More interestingly, the resulting crystalline CTF-1 also showed high photocatalytic performance for oxygen evolution. Because photocatalytic oxygen evolution has a slower dynamic than hydrogen evolution, it is challenging to find effective photocatalysts. Wang et al. recently found that CTFs can be a good platform to tune the band structures and achieve optimal photocatalytic oxygen evolution rates. They showed that the variation of the length of the monomers can modulate the properties of CTFs for oxygen evolution and therefore provided a new strategy to design CTFs for oxygen photocatalysis [78].

**Figure 3:**
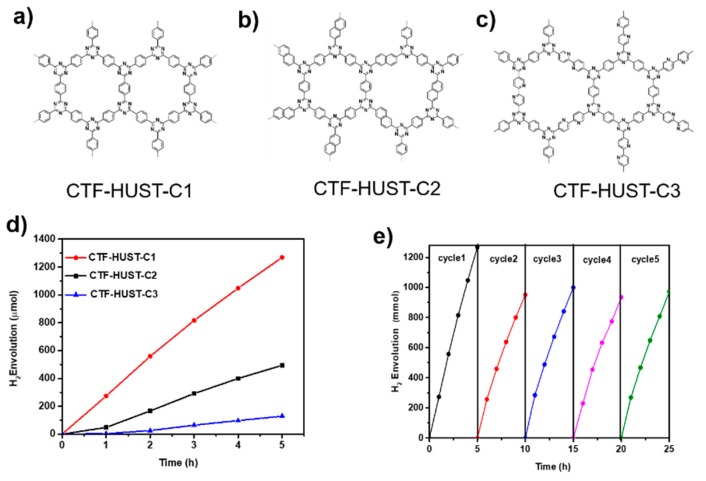
(**a**–**c**) Structure of highly crystalline CTFs obtained by the polycondensation method, (**d**) time course of photocatalytic hydrogen evolution and (**e**) recycle experiment of a CTF-HUST-C1 sample. (adapted from reference 79). Reprinted with permission from [79]. Copyright 2018, Wiley-VCH.

Since most of the present CTFs are synthesized by trimerization of nitrile building blocks, structure diversity is somewhat limited. We recently developed CTFs synthesized by polycondensation showing high photocatalytic performance for hydrogen evolution as compared to many reported CTFs [12]. The high catalytic activity can be attributed to the layered morphologies and crystalline structures of the resulting CTFs. We further found that photocatalytic hydrogen evolution can be dramatically improved by increasing the crystallinity of CTFs (Figure 3) [79]. We expect that the design and synthesis of CTFs by polycondensation will result in much higher photocatalytic performance and great promising applications.

#### 3.2.2. Photocatalytic Carbon Dioxide Reduction

**Figure 4:**
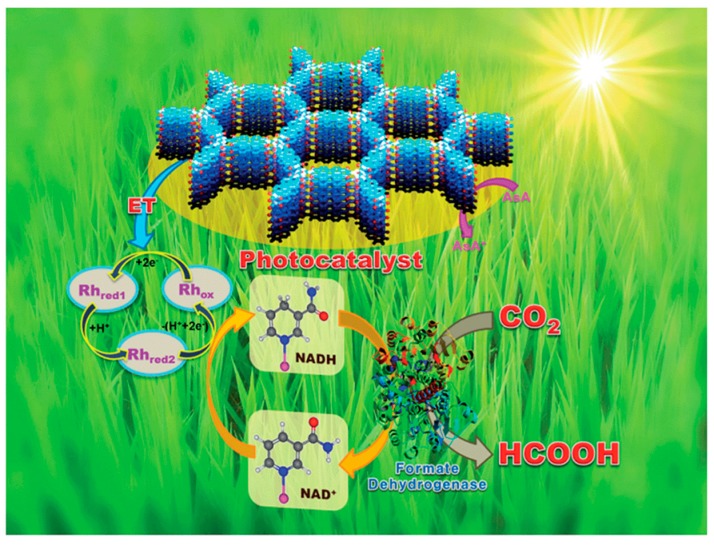
Photocatalytic carbon dioxide reduction to formic acid by CTFs. Reprinted with permission from [80]. Copyright 2016, The Royal Society of Chemistry.

In addition to hydrogen evolution, CTFs were also reported to be potential photocatalysts for carbon dioxide conversion into value-added fuels. In 2016, Baeg et al. firstly reported 2D CTFs based on the condensation of cyanuric chloride and perylenediimide monomers (Figure 4) [80], which were prepared into a film and applied for the photocatalytic reduction of carbon dioxide for the first time. The results showed that formic acid could be produced from the reduction of CO_2_ by artificial photosynthesis. They reported that the CTF photocatalyst could have an activity of 881.3 × 106 nmol g cat^−1^ h^−1^, which is about 3.7 times higher than that of films fabricated only with the monomer. The CTF photocatalyst also showed a good stability which and could be reused for three cycles. In addition, CTFs can allow post-modifications and function as photocatalysts. For example, Cao et al. recently reported that a CTF modified with Rhenium can work as an efficient photocatalyst for CO_2_ conversion into CO [42]. They found that the photocatalyst could achieve a CO evolution rate as high as 353 μmol h^−1^ g^−1^ in a solid–gas system [42]. This solid–gas system and supported catalysts have advantages in avoiding of dimerization and leaching of Re species which were proposed to cause loss of the activity.

### 3.3. Electrocatalysis for Energy Storage and Conversion

#### 3.3.1. Oxygen Reduction and Methane Oxidation

**Figure 5:**
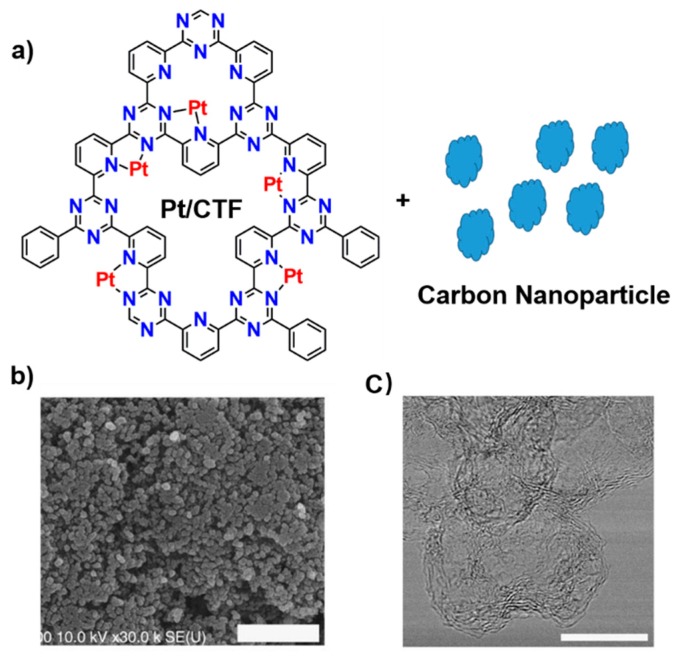
(**a**) Structure of Pt–CTF–CP (carbon particles), (**b**) SEM image and (**c**) TEM image of Pt–CTF–CP (Adapted from Reference 30) [30] (Copyright, Nature Publishing Group).

CTFs are abundant in nitrogen. In addition, the building blocks are designable. Therefore, CTFs have promising applications in electrocatalysts. However, one of the disadvantages of CTFs for electrocatalysis is their low conductivity. To mitigate this deficiency, Nakanishi et al. developed a new strategy to prepare CTFs in the presence of carbon nanoparticles (Figure 5) [30]. By this strategy, the conductivity was improved, and the electrocatalyst was prepared by supporting platinum on the CTF. They successfully applied it in an oxygen reduction reaction and found that the resulting new material was inactive in methanol oxidation and thus is a promising cathode material in methanol fuel cells. Beside precious metals, non-precious metals, such as copper, can also be introduced to enhance oxygen reduction reaction (ORR) performance. Kamiya et al. showed copper-modified CTFs together with carbon nanoparticles are good electrocatalysts [81]. The onset potential of the resulting electrode material is as high as 810 mV (vs. RHE, pH 7), which is the highest value for copper-based electrocatalysts in neutral conditions reported so far in the literature. 

**Figure 6:**
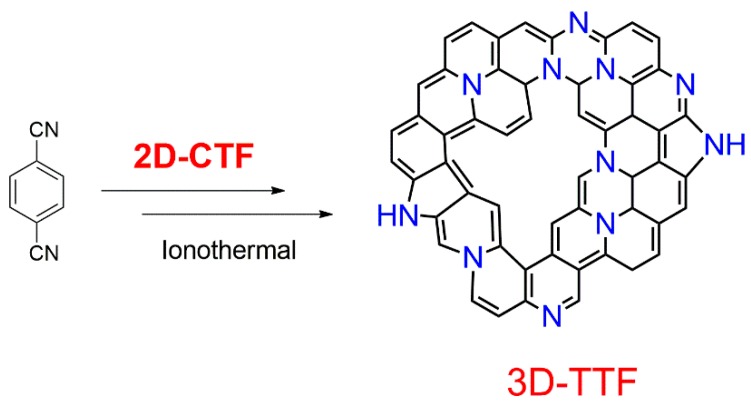
Bottom-up strategy to prepare 3D TTF (thermalized triazine-based framework) catalysts for oxygen reduction reaction by trimerization of terephthalonitrile at high temperatures (adapted from [82]). Reprinted with permission from [82]. Copyright 2015, Wiley-VCH.

Besides allowing modifications of metal sites in the electrocatalysts, CTFs may also serve as metal-free electrocatalysts. For example, Hao et al. developed a bottom-up approach to construct CTFs as metal-free electrocatalysts for ORR (Figure 6) [82]. They synthesized CTF-1 at different temperatures, which allowed them to adjust the nitrogen content as well as the conductivity of the materials. They further showed that other heteroatoms such as B or F could be incorporated in the CTF, and different heteroatoms conferred disparate activities. When B was introduced in the CTF, a good selectivity was obtained, while F gave a higher activity [82]. 

The same strategy can be used to prepare metal complex-modified catalysts in other oxidation reactions, such as methane oxidation to methanol. The work by Schüth et al. developed a type of solid catalysts which can selectively oxidize methane to methanol at a low temperature. The stable catalysts complexed with Pt show a high activity with a TON of 300 even after five catalytic cycles [83]. Recently, these authors further studied the effect of the local platinum environments in solid catalysts on the catalytic performance in methane oxidation in the presence of sulfuric acid and compared it to that of the molecular Periana catalyst [84]. These results demonstrate that CTFs are a powerful and potential platform for the design of supported catalysts for various useful transformations.

#### 3.3.2. Supercapactiors and Batteries

The research of the use of organic materials in energy storage applications, such as for supercapacitors and batteries, is an important task. In organic materials for supercapacitor applications, the heteroatom and surface area are important factors to be considered for achieving high performance in energy storage. In these regards, the nitrogen-rich structure of CTFs is a particular interesting platform for supercapacitors because they have high nitrogen content and large surface areas, and these factors vary considerably.

**Figure 7:**
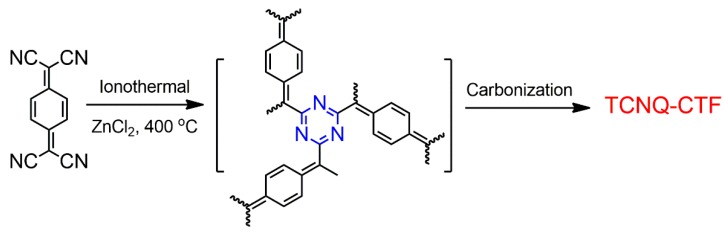
Procedure for the preparation of TCNQ–CTF [58].

Zhi et al. studied the structural evolution of high-performance energy storage for supercapacitors in CTFs [45,85]. They selected 2D CTFs with microporous structures and investigated the correlation of nitrogen content and micropores with supercapacitance performance. It was revealed that not only the heteroatom affects the performance, but also the micropores may contribute to a high performance. Zhu et al. developed a new strategy to construct CTF-1 and graphene hybridized materials with a sandwich-type structure, which can maintain a high surface area and a two-dimensional morphology [46]. The resulting materials exhibit excellent supercapacitor performance compared to porous carbon materials. Deng et al. recently reported a series of highly porous CTFs (TCNQ–CTFs) based on 7,7,8,8-tetracyanoquinodimethane (TCNQ) building blocks (Figure 7) [58]. TCNQ–CTFs were prepared at different temperatures varying from 400 to 900 °C at ionothermal conditions. They found that TCNQ–CTF-800 provided the highest nitrogen content of 8.13% and the highest BET surface area of 3663 m^2^ g^−1^. The supercapacitance measurement showed that a high capacitance of 383 F g^−1^ was achieved in alkaline conditions. These results may provide principles for the future design and synthesis of materials. The importance of the heteroatom, the pore size distribution in the materials, and the choice of the building blocks need to be considered in the design of materials for supercapacitors.

CTFs were also developed as electrode materials for metal ion batteries by using their nitrogen-rich and high surface area structures. Wang et al. reported that CTF-1 is a potential material to trap polysulfide, and thus the resulting cathode materials have a positive effect on the retention performance of lithium–sulfur batteries [86]. CTF-1/S@155 °C could even exhibit a high capacity of 541 mA h g^−1^ at a high current density of 1 C. The good performance was explained by the crystalline and nanoporous structure of CTF-1, which increased the conductivity of sulfur and allowed the transportation of the electrolyte. Coskun et al. also developed a sulfur-doped CTF, which was synthesized by the elemental sulfur-mediated method. The resulted S-CTF-1 exhibited a high performance and cyclic stability. It could retain 90.6, 83.9, 76.1, 68.7, and 59.8% of the initial capacity (670 mAhg^−1^) at 0.1 C, 0.2 C, 0.5 C, 1 C, and 2 C, respectively [87]. Shan et al. further showed that incorporation of graphene into the CTFs could be a promising strategy to improve the charge transfer and electronic conduction in the lithium–sulfur battery. They found that graphene-directed 2D nitrogen doped porous carbon framework (GPF)–sulfur nanocomposites exhibited a capacity as high as 962 mA h g^−1^ at 2 A g^−1^ and even a high capacity of 591 mA h g^−1^ at 20 A g^−1^ [88]. On the other hand, the separator is an important element that influences lithium–sulfur batteries. For this purpose, Stoeck et al. implemented CTF-1 as a conductive separator coating. Due to its nitrogen-rich and porous structure, it enhanced the cell performance, with the capacity improving by 2.5 times from 405 to 1016 mAh g^−1^ at C/5 charging rate [89]. By using the same method developed by Coskun and coworkers, Xu et al. demonstrated that fluorinated CTFs can by prepared with sulfur and show much higher capacity than non-fluorinated CTF in lithium–sulfur batteries. They concluded that introducing fluorine supplies strong anchoring sites, which can alleviate the dissolution and migration of polysulfides [90].

#### 3.3.3. Electrocatalytic Carbon Dioxide Reduction

**Figure 8:**
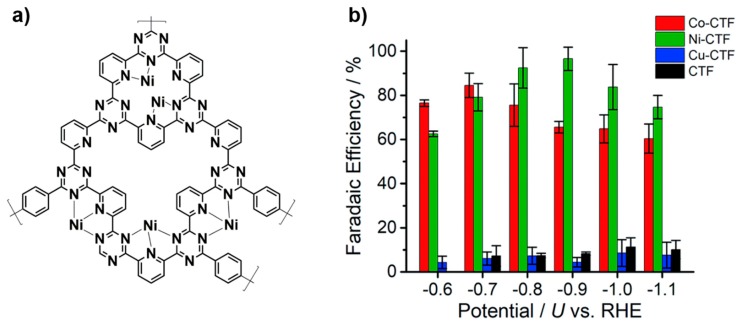
(**a**) Representative Structure of Ni–CTFs. (**b**) The faradaic efficiency of different metal coordinated CTFs at different potentials. (Adapted from reference 91) [91] (Copyright 2018, The Royal Society of Chemistry).

The electrocatalytic conversion of CO_2_ to useful chemicals or value-added chemicals is also a promising route to address environmental and energy issues. However, most materials have still low efficiencies, such as low faradaic efficiencies and large overpotentials. Recently, using CTFs in electrocatalysis to reduce CO_2_ has received attention. Su et al. have adopted a pyridine-containing CTF to work as ab efficient electrocatalsyt for CO_2_ reduction to CO (Figure 8) [91]. They introduced a pyridine unit in the CTF and provided coordination sites for various metals, i.e., Co, Ni, Cu. Then, they investigated the influence of different metal atoms on the electrocatalysis performance. It was shown that Ni–CTF gave the best performance as compared to other metals. It also showed better performance than metal-porphyin structures. They further used theoretical calculation to reveal the mechanism of the enhanced performance, and attributed it to the low coordination of the CTF structure, which gave lower free-energy barriers for the adsorption of COOH on the coordination sites [91].

The selectivity of the products is an important factor that is meaningful to the catalytic performance. More recently, Wang et al. developed a new strategy which introduced abundant fluorine in the CTF structure for electrocatalytic CO_2_ reduction to methane [92]. They found that the high selectivity was ascribed to fluorine which affected N activity for CH_4_ evolution, by an electrochemical study and a theoretical investigation. Therefore, CTFs could be useful platforms to introduce heteroatoms, halogen elements, or metal sites for a high electrocatalysis performance in CO_2_ reduction. These strategies may provide opportunities for the design and synthesis of efficient photocatalysts for CO_2_ reduction.

## 4. Outlook 

In summary, CTFs are a promising platform to create a variety of materials to address environment and energy issues by using their intrinsic nitrogen-rich scaffold and conjugated semiconductive structures. Recent advancements have shown that new synthesis methods are becoming available for more structural designs and practical application. Particularly, CTFs have shown an enormous potential for water splitting and carbon dioxide reduction, irrespective of the use photocatalysis or electrocatalysis. Thus, we can expect that the application of CTFs in the environment and energy fields will become more widespread. However, we also face some challenges. Once their synthesis is achieved, how to further enhance their surface area for gas adsorption and storage and how to develop a more general strategy to obtain high-crystalline CTFs for photocatalysis or electrocatalysis applications remain open questions. These questions still impel us to find new solutions. For energy- and environment-related applications, performance can largely be improved, and this goal will provide future research opportunities.

## Figures and Tables

**Table 1:** Summary of the recent results of hydrogen uptake reported in the literature. CTFs: covalent triazine frameworks.

CTFs	Surface Area (m^2^ g^−1^)	Condition	Hydrogen Uptake (wt %)	References
DCBP network	2475	1.00 bar, 77 K	1.55	[6]
TPC-2	1250	1.00 bar, 77 K	2.34	[60]
TPC-3	1530	1.00 bar, 77 K	1.84	[60]
PCTF-1	2235	1.0 bar, 77 K	1.86	[61]
fl-CTF400	2862	1.0 bar, 77 K	1.95	[62]
fl-CTF400	2862	20 bar, 77 K	4.36	[62]
caCTF-1-700	2367	77, 1 bar	2.46	[26]
caCTF-1-700	2367	87 K, 1 bar	1.66	[26]
CTF–Li6	-	-	12.3	[63]
CTF–Na6	-	-	10.3	[63]
CTF–Ca6	-	-	8.8	[63]

Notes: DCBP: 4,4-dicycnobiphenyl; TPC: triazine-based porous carbon; fl: fluorine; ca: chemical activation.

**Table 2:** Summary of the results of the CO_2_ uptake reported in the literatures showing the recent progress in the field. Acac-CTF: acetylacetone CTF.

CTFs	Surface Area (m^2^ g^−1^)	Condition	CO_2_ Uptake	References
P6M	947	273 K and 1 bar	4.17 mmol g^−1^	[10]
PCTF-1	2235	273 K, 1 bar	73.0 cm g^−1^	[65]
PCTF-5	1183	273 K, 1 bar	58.1 cm g^−1^	[65]
PCTF-7	613	273 K, 1 bar	48.9 cm g^−1^	[65]
CTF-1-600	1553	273 K, 1 bar	3.83 mmol g^−1^	[66]
F-CTF-1-600	1535	273 K, 1 bar	5.53 mmol g^−1^	[66]
pym-CTF-500	208	273 K, 1 bar	2.75 mmol g^−1^	[67]
Bipy-CTF600	2479	273 K, 1 bar	5.58 mmol g^−1^	[67]
Ad4L1	1617	273 K, 1 bar	76.33 cm^3^ g^−1^	[68]
Ad4L3	1341	273 K, 1 bar	74.58 cm^3^ g^−1^	[68]
MM1	1800	273 K, 1 bar	83.5 cm^3^ g^−1^ (3.68 mmol g^−1^)	[69]
MM2	1360	273 K, 1 bar	106.8 cm^3^ g^−1^ (4.70 mmol g^−1^)	[69]
CTF-Ph	1991	273 K, 1 bar	3.05 mmol g^−1^	[70]
CTF-Py	1239	273 K, 1 bar	3.79 mmol g^−1^	[70]
CTF-20-400	1458	273 K, 1 bar	3.48 mmol g^−1^	[71]
CTF-5-500	853	273 K, 1 bar	3.02 mmol g^−1^	[71]
CTF-CSU1	685	273 K, 1 bar	15.1 wt %	[72]
CTF-CSU19	982	273 K, 1 bar	12.9 wt %	[72]
Acac-CTF-10-500	1556	273 K, 1 bar	3.30 mmol g^−1^	[55]
TPC-1	1940	273 K, 1 bar	4.90 mmol g^−1^	[73]
cCTF-400	744	273 K, 1 bar	126 mg g^−1^	[26]
cCTF-500	1247	273 K, 1 bar	133 mg g^−1^	[26]
TRIPTA-COF	609	273 K, 5 bar	12.97 mmol g^−1^	[74]

Notes: Pym-pyrimidine; Bipy-bipyridine; TRIPTA-1,3,5-tris(4-aminophenyl)triazine.

## References

[1] Das S., Heasman P., Ben T., Qiu S.L. (2017). Porous organic materials: Strategic design and structure-function correlation. Chem. Rev..

[2] Wu D.C., Xu F., Sun B., Fu R.W., He H.K., Matyjaszewski K. (2012). Design and preparation of porous polymers. Chem. Rev..

[3] Xu Y.H., Jin S.B., Xu H., Nagai A., Jiang D.L. (2013). Conjugated microporous polymers: Design, synthesis and application. Chem. Soc. Rev..

[4] Ding S.Y., Wang W. (2013). Covalent organic frameworks (COFs): From design to applications. Chem. Soc. Rev..

[5] Feng X., Ding X.S., Jiang D.L. (2012). Covalent organic frameworks. Chem. Soc. Rev..

[6] Kuhn P., Antonietti M., Thomas A. (2008). Porous, covalent triazine-based frameworks prepared by ionothermal synthesis. Angew. Chem. Int. Ed..

[7] Ben T., Qiu S.L. (2013). Porous aromatic frameworks: Synthesis, structure and functions. CrystEngComm.

[8] Tsyurupa M.P., Davankov V.A. (2002). Hypercrosslinked polymers: basic principle of preparing the new class of polymeric materials. React. Funct. Polym..

[9] McKeown N.B., Budd P.M., Msayib K.J., Ghanem B.S., Kingston H.J., Tattershall C.E., Makhseed S., Reynolds K.J., Fritsch D. (2005). Polymers of intrinsic microporosity (PIMs): Bridging the void between microporous and polymeric materials. Chem. Eur. J..

[10] Ren S., Bojdys M.J., Dawson R., Laybourn A., Khimyak Y.Z., Adams D.J., Cooper A.I. (2012). Porous, fluorescent, covalent triazine-based frameworks via room-temperature and microwave-assisted synthesis. Adv. Mater..

[11] Meier C.B., Sprick R.S., Monti A., Guiglion P., Lee J.M., Zwijnenburg M.A., Cooper A.I. (2017). Structure-property relationships for covalent triazine-based frameworks: The effect of spacer length on photocatalytic hydrogen evolution from water. Polymer.

[12] Wang K.W., Yang L.M., Wang X., Guo L.P., Cheng G., Zhang C., Jin S.B., Tan B., Cooper A. (2017). Covalent triazine frameworks via a low-temperature polycondensation approach. Angew. Chem. Int. Ed..

[13] Zhu X., Tian C., Mahurin S.M., Chai S.H., Wang C., Brown S., Veith G.M., Luo H., Liu H., Dai S. (2012). A Superacid-catalyzed synthesis of porous membranes based on triazine frameworks for CO_2_ separation. J. Am. Chem. Soc..

[14] Katekemol P., Roeser J., Bojdys M.J., Weber J., Thomas A. (2013). Covalent triazine frameworks prepared from 1,3,5-tricyanobenzene. Chem. Mater..

[15] Gomes R., Bhanja P., Bhaumik A. (2015). A triazine-based covalent organic polymer for efficient CO_2_ adsorption. Chem. Commun..

[16] Gu C.Y., Liu D.Y., Huang W., Liu J., Yang R.Q. (2015). Synthesis of covalent triazine-based frameworks with high CO_2_ adsorption and selectivity. Polym. Chem..

[17] Puthiaraj P., Cho S.M., Lee Y.R., Ahn W.S. (2015). Microporous covalent triazine polymers: Efficient Friedel-Crafts synthesis and adsorption/storage of CO_2_ and CH_4_. J. Mater. Chem. A.

[18] Saleh M., Baek S.B., Lee H.M., Kim K.S. (2015). Triazine-based microporous polymers for selective adsorption of CO_2_. J. Phys. Chem. C.

[19] Bhunia A., Esquivel D., Dey S., Fernandez-Teran R., Goto Y., Inagaki S., Voort P.V.D., Janiak C. (2016). A photoluminescent covalent triazine framework: CO_2_ adsorption, light-driven hydrogen evolution and sensing of nitroaromatics. J. Mater. Chem. A.

[20] Dey S., Bhunia A., Esquivelb D., Janiak C. (2016). Covalent triazine-based frameworks (CTFs) from triptycene and fluorene motifs for CO_2_ adsorption. J. Mater. Chem. A.

[21] Puthiaraj P., Kim S.S., Ahn W.S. (2016). Covalent triazine polymers using a cyanuric chloride precursor via Friedel-Crafts reaction for CO_2_ adsorption/separation. Chem. Eng. J..

[22] Tao L.M., Niu F., Liu J.G., Wang T.M., Wang Q.H. (2016). Troger’s base functionalized covalent triazine frameworks for CO_2_ capture. RSC Adv..

[23] Tao L.M., Niu F., Wang C., Liu J.G., Wang T.M., Wang Q.H. (2016). Benzimidazole functionalized covalent triazine frameworks for CO_2_ capture. J. Mater. Chem. A.

[24] Wang K., Huang H., Liu D., Wang C., Li J., Zhong C. (2016). Covalent triazine-based frameworks with ultramicropores and high nitrogen contents for highly selective CO_2_ capture. Environ. Sci. Technol..

[25] Zhu X., Tian C., Veith G.M., Abney C.W., Dehaudt J., Dai S. (2016). In situ doping strategy for the preparation of conjugated triazine frameworks displaying efficient CO_2_ capture performance. J. Am. Chem. Soc..

[26] Lee Y.J., Naidu S.N., Coskun A. (2017). Chemically activated covalent triazine frameworks with enhanced textural properties for high capacity gas storage. ACS Appl. Mater. Interf..

[27] Buyukcakir O., Je S.H., Talapaneni S.N., Kim D., Coskun A. (2017). Charged covalent triazine frameworks for CO_2_ capture and conversion. ACS Appl. Mater. Interf..

[28] Yuan K.Y., Liu C., Han J., Yu G.P., Wang J., Duan H., Wang Z., Jian X.G. (2016). Phthalazinone structure-based covalent triazine frameworks and their gas adsorption and separation properties. RSC Adv..

[29] Liebl M.R., Senker J. (2013). Microporous functionalized triazine-based polyimides with high CO_2_ capture capacity. Chem. Mater..

[30] Kamiya K., Kamai R., Hashimoto K., Nakanishi S. (2014). Platinum-modified covalent triazine frameworks hybridized with carbon nanoparticles as methanol-tolerant oxygen reduction electrocatalysts. Nat. Commun..

[31] Artz J., Mallmann S., Palkovits R. (2015). Selective aerobic oxidation of HMF to 2,5-Diformylfuran on covalent triazine frameworks-supported Ru catalysts. ChemSusChem.

[32] Chan-Thaw C.E., Villa A., Wang D., Santo V.D., Biroli A.O., Veith G.M., Thomas M., Prati L. (2015). PdHx entrapped in a covalent triazine framework modulates selectivity in glycerol oxidation. ChemCatChem.

[33] Chan-Thaw C.E., Villa A., Katekomo P., Su D., Thomas A., Prati L. (2010). Covalent Triazine Framework as Catalytic Support for Liquid Phase Reaction. Nano Lett..

[34] Puthiaraj P., Lee Y.R., Zhang S.Q., Ahn W.S. (2016). Triazine-based covalent organic polymers: Design, synthesis and applications in heterogeneous catalysis. J. Mater. Chem. A.

[35] Roeser J., Kailasam K., Thomas A. (2012). Covalent triazine frameworks as heterogeneous catalysts for the synthesis of cyclic and linear carbonates from carbon dioxide and epoxides. ChemSusChem.

[36] Gunasekar G.H., Park K., Ganesan V., Lee K., Kim N.K., Jung K.D., Yoon S. (2017). A covalent triazine framework, functionalized with Ir/N-heterocyclic carbene sites, for the efficient hydrogenation of CO_2_ to formate. Chem. Mater..

[37] Artz J. (2018). Covalent triazine-based frameworks tailor-made catalysts and catalyst supports for molecular and nanoparticulate species. ChemCatChem.

[38] Cui Y.Z., Du J.F., Liu Y.C., Yu Y., Wang S., Pang H., Liang Z.Q., Yu G.H. (2018). Design and synthesis of a multifunctional porous N-rich polymer containing s-triazine and Troger’s base for CO_2_ adsorption, catalysis and sensing. Polym. Chem..

[39] Iwase K., Kamiya K., Miyayama M., Hashimoto K., Nakanishi S. (2018). Sulfur-linked covalent triazine frameworks doped with coordinatively unsaturated Cu(I) as electrocatalysts for oxygen reduction. ChemElectroChem..

[40] Kann A., Hartmann H., Besmehn A., Hausoul P.J.C., Palkovits R. (2018). Hydrogenation of CO_2_ to formate over ruthenium immobilized on solid molecular rhosphines. ChemSusChem.

[41] Xu N., Wang R.L., Li D.P., Meng X., Mu J.L., Zhou Z.Y., Su Z.M. (2018). A new triazine-based covalent organic polymer for efficient photodegradation of both acidic and basic dyes under visible light. Dalton Trans..

[42] Xu R., Wang X.S., Zhao H., Lin H., Huang Y.B., Cao R. (2018). Rhenium-modified porous covalent triazine framework for highly efficient photocatalytic carbon dioxide reduction in a solid-gas system. Catal. Sci. Technol..

[43] Zhu G., Shi S., Liu M., Zhao L., Wang M., Zheng X., Gao J., Xu J. (2018). Formation of strong basicity on covalent triazine frameworks as catalysts for the oxidation of methylene compounds. ACS Appl. Mater. Interfaces.

[44] Hao L., Li X.L., Zhi L.J. (2013). Carbonaceous electrode materials for supercapacitors. Adv. Mater..

[45] Hao L., Ning J., Luo B., Wang B., Zhang Y., Tang Z., Yang J., Thomas A., Zhi L. (2015). Structural evolution of 2D microporous covalent triazine-based framework toward the study of high-performance supercapacitors. J. Am. Chem. Soc..

[46] Zhu J.H., Zhuang X.D., Yang J., Feng X.L., Hiranod S. (2017). Graphene-coupled nitrogen-enriched porous carbon nanosheets for energy storage. J. Mater. Chem. A.

[47] Bhanja P., Das S.K., Bhunia K., Pradhan D., Hayashi T., Hijikata Y., Irle S., Bhaumik A. (2018). A new porous polymer for highly efficient capacitive energy storage. ACS Sustain. Chem. Eng..

[48] Bi J.H., Fang W., Li L.Y., Wang J.Y., Liang S.J., He Y.H., Liu M.H., Wu L. (2015). Covalent triazine-based frameworks as visible light photocatalysts for the splitting of water. Macromol. Rapid Commun..

[49] Schwinghammer K., Hug S., Mesch M.B., Senkerd J., Lotsch B.V. (2015). Phenyl-triazine oligomers for light-driven hydrogen evolution. Energy Environ. Sci..

[50] Huang W., Byun J., Rörich I., Ramanan C., Blom P.W.M., Lu H., Wang D., da Caire S.L., Li R., Wang L. (2018). Asymmetric covalent triazine framework for enhanced visible-light photoredox catalysis via energy transfer cascade. Angew. Chem. Int. Ed..

[51] Jiang Q., Sun L., Bi J., Liang S., Li L., Yu Y., Wu L. (2018). MoS_2_ quantum dots-modified covalent triazine-based frameworks for enhanced photocatalytic hydrogen evolution. ChemSusChem..

[52] Huang W., Wang Z.J., Ma B.C., Ghasimi S., Gehrig D., Laquai F., Landfester K., Zhang K. (2016). Hollow nanoporous covalent triazine frameworks via acid vapor-assisted solid phase synthesis for enhanced visible light photoactivity. J. Mater. Chem. A.

[53] Hug S., Tauchert M.E., Li S., Pachmayr U.E., Lotsch B.V. (2012). A functional triazine framework based on N-heterocyclic building blocks. J. Mater. Chem..

[54] Ma H., Ren H., Meng S., Sun F., Zhu G. (2013). Novel porphyrinic porous organic frameworks for high performance separation of small hydrocarbons. Sci. Rep..

[55] Jena H.S., Krishnara C., Wang G., Leus K., Schmidt J., Chaoui N., Voort P.V.D. (2018). Acetylacetone covalent triazine framework: An efficient carbon capture and storage material and a highly stable heterogeneous catalyst. Chem. Mater..

[56] Liu J.J., Zan Z., Li L., Yang Y., Bu F.X., Xu Y.X. (2017). Solution synthesis of semiconducting two-dimensional polymer via trimerization of carbonitrile. J. Am. Chem. Soc..

[57] Yu S.Y., Mahmood J., Noh H.J., Seo J.M., Jung S.M., Shin S.H., Im Y.K., Jeon I.Y., Baek J.B. (2018). Direct synthesis of a covalent triazine-based framework from aromatic amides. Angew. Chem. Int. Ed..

[58] Li Y.J., Zheng S.H., Liu X., Li P., Sun L., Yang R.X., Wang S., Wu Z.S., Bao X.H., Deng W.Q. (2018). Conductive microporous covalent triazine-Based framework for high-performance electrochemical capacitive energy storage. Angew. Chem. Int. Ed..

[59] Xie J.J., Shevlin S.A., Ruan Q.S., Moniz S.J.A., Liu Y.R., Liu X., Li Y.M., Lau C.C., Guo Z.X., Tang J.W. (2018). Efficient visible light-driven water oxidation and proton reduction by an ordered covalent triazine-based framework. Energy Environ. Sci..

[60] Hu X.M., Chen Q., Zhao Y.C., Laursen B.W., Han B.H. (2016). Facile synthesis of hierarchical triazine-based porous carbons for hydrogen storage. Microporous Microporous Mater..

[61] Bhunia A., Vasylyeva V., Janiak C. (2013). From a supramolecular tetranitrile to a porous covalent triazine-based framework with high gas uptake capacities. Chem. Commun..

[62] Hug S., Mesch M.B., Oh H., Popp N., Hirscher M., Senker J., Lotsch B.V. (2014). A fluorene based covalent triazine framework with high CO_2_ and H_2_ capture and storage capacities. J. Mater. Chem. A.

[63] Chen X.W., Yuan F., Gu Q.F., Yu X.B. (2013). Light metals decorated covalent triazine-based frameworks as a high capacity hydrogen storage medium. J. Mater. Chem. A.

[64] He H., Chen X., Zou W., Li R. (2018). Transition metal decorated covalent triazine-based frameworks as a capacity hydrogen storage medium. Int. J. Hydrogen. Energ..

[65] Bhunia A., Boldog I., Moller A., Janiak C. (2013). Highly stable nanoporous covalent triazine-based frameworks with an adamantane core for carbon dioxide sorption and separation. J. Mater. Chem. A.

[66] Zhao Y.F., Yao K.X., Teng B.Y., Zhang T., Han Y. (2013). A perfluorinated covalent triazine-based framework for highly selective and water-tolerant CO_2_ capture. Energy Environ. Sci..

[67] Hug S., Stegbauer L., Oh H., Hirscher M., Lotsch B.V. (2015). Nitrogen-rich covalent triazine frameworks as high-performance platforms for selective carbon capture and storage. Chem. Mater..

[68] Dey S., Bhunia A., Boldog I., Janiak C. (2017). A mixed-linker approach towards improving covalent triazine-based frameworks for CO_2_ capture and separation. Microporous Microporous Mater..

[69] Dey S., Bhunia A., Breitzke H., Groszewicz P.B., Buntkowskyb G., Janiak C. (2017). Two linkers are better than one: Enhancing CO_2_ capture and separation with porous covalent triazine-based frameworks from mixed nitrile linkers. J. Mater. Chem. A..

[70] Tuci G., Pilaski M., Ba H., Rossin A., Luconi L., Caporali S., Pham-Huu C., Palkovits R., Giambastiani G. (2017). Unraveling surface basicity and bulk morphology relationship on covalent triazine frameworks with unique catalytic and gas adsorption properties. Adv. Funct. Mater..

[71] Wang G., Leus K., Zhao S., Voort P.V.D. (2018). Newly designed covalent triazine framework based on novel N-heteroaromatic building blocks for efficient CO_2_ and H_2_ capture and storage. ACS Appl. Mater. Interfaces.

[72] Yu W., Gu S., Fu Y., Xiong S., Pan C., Liu Y., Yu G. (2018). Carbazole-decorated covalent triazine frameworks: Novel nonmetal catalysts for carbon dioxide fixation and oxygen reduction reaction. J. Catal..

[73] Hu X.M., Chen Q., Zhao Y.C., Laursen B.W., Han B.H. (2014). Straightforward synthesis of a triazine-based porous carbon with high gas-uptake capacities. J. Mater. Chem. A.

[74] Gomes R., Bhaumik A. (2016). A new triazine functionalized luminescent covalent organic framework for nitroaromatic sensing and CO_2_ storage. RSC Adv..

[75] Jiang X., Wang P., Zhao J.J. (2015). 2D covalent triazine framework: A new class of organic photocatalyst for water splitting. J. Mater. Chem. A.

[76] Li L., Fang W., Zhang P., Bi J., He Y., Wang J., Su W. (2016). Sulfur-doped covalent triazine-based frameworks for enhanced photocatalytic hydrogen evolution from water under visible light. J. Mater. Chem. A.

[77] Kuecken S., Acharjya A., Zhi L., Schwarze M., Schomäcker R., Thomas A. (2017). Fast tuning of covalent triazine frameworks for photocatalytic hydrogen evolution. Chem. Commun..

[78] Lan Z.A., Fang Y., Zhang Y., Wang X. (2018). Photocatalytic oxygen evolution from functional triazine-based polymers with tunable band structures. Angew. Chem. Int. Ed..

[79] Liu M.Y., Huang Q., Wang S.L., Li Z.Y., Li B.Y., Jin S.B., Tan B. (2018). Crystalline covalent triazine frameworks by in situ oxidation of alcohols to aldehyde monomers. Angew. Chem. Int. Ed..

[80] Yadav R.K., Kumar A., Park N.J., Kong K.J., Baeg J.O. (2016). A highly efficient covalent organic framework film photocatalyst for selective solar fuel production from CO_2_. J. Mater. Chem. A.

[81] Iwase K., Yoshioka T., Nakanishi S., Hashimoto K., Kamiya K. (2015). Copper-modified covalent triazine frameworks as non-noble-metal electrocatalysts for oxygen reduction. Angew. Chem. Int. Ed..

[82] Hao L., Zhang S.S., Liu R.J., Ning J., Zhang G.J., Zhi L.J. (2015). Bottom-up construction of triazine-based frameworks as metal-free electrocatalysts for oxygen reduction reaction. Adv. Mater..

[83] Palkovits R., Antonietti M., Kuhn P., Thomas A., Schüth F. (2009). Solid catalysts for the selective low-temperature oxidation of methane to methanol. Angew. Chem. Int. Ed..

[84] Soorholtz M., Jones L.C., Samuelis D., Weidenthaler C., White R.J., Titirici M.M., Cullen D.A., Zimmermann T., Antonietti M., Maier J. (2016). Local platinum environments in a solid analogue of the molecular periana catalyst. ACS Catal..

[85] Hao L., Luo B., Li X.L., Jin M.H., Fang Y., Tang Z.H., Jia Y.Y., Liang M.H., Thomas A., Yang J.H. (2012). Terephthalonitrile-derived nitrogen-rich networks for high performance supercapacitors. Energy Environ. Sci..

[86] Liao H., Ding H., Li B., Ai X., Wang C. (2014). Covalent-organic frameworks: Potential host materials for sulfur impregnation in lithium–sulfur batteries. J. Mater. Chem. A.

[87] Talapaneni S.N., Hwang T.H., Je S.H., Buyukcakir O., Choi J.W., Coskun A. (2016). elemental-sulfur-mediated facile synthesis of a covalent triazine framework for high-performance lithium–sulfur batteries. Angew. Chem. Int. Ed..

[88] Shan J., Liu Y., Su Y., Liu P., Zhuang X., Wu D., Zhang F., Feng X. (2016). Graphene-directed two-dimensional porous carbon frameworks for high-performance lithium–sulfur battery cathodes. J. Mater. Chem. A.

[89] Stoeck U., Balach J., Klose M., Wadewitz D., Ahrens E., Eckert J., Giebeler L. (2016). Reconfiguration of lithium sulphur batteries: “Enhancement of Li–S cell performance by employing a highly porous conductive separator coating”. J. Power Sources.

[90] Xu F., Yang S., Jiang G., Ye Q., Wei B., Wang H. (2017). Fluorinated, sulfur-rich, covalent triazine frameworks for enhanced confinement of polysulfides in lithium–sulfur batteries. ACS Appl. Mater. Interfaces.

[91] Su P., Iwase K., Harada T., Kamiya K., Nakanishi S. (2018). Covalent triazine framework modified with coordinatively-unsaturated Co or Ni atoms for CO_2_ electrochemical reduction. Chem. Sci..

[92] Wang Y.S., Chen J.X., Wang G.X., Li Y., Wen Z.H. (2018). Perfluorinated covalent triazine framework derived hybrids for the highly selective electroconversion of carbon dioxide into methane. Angew. Chem. Int. Ed..

